# Impact of serum calcium levels on the occurrence of sepsis and prognosis in hospitalized patients with concomitant psoriasis: a retrospective study based on the MIMIC-IV database

**DOI:** 10.3389/fimmu.2025.1621231

**Published:** 2025-07-22

**Authors:** Xiaolong Zheng, Qianjin Su, Yedi Wang, Xuefeng Geng

**Affiliations:** ^1^ Department of Orthopedics, the 963rd Hospital of the Joint Service Support Force of the PLA, Jiamusi, China; ^2^ Department of Dermatology, the 963rd Hospital of the Joint Service Support Force of the PLA, Jiamusi, China

**Keywords:** psoriasis, serum calcium, mediation analysis, sepsis, machine learning, trajectory modeling

## Abstract

**Objective:**

This study aims to investigate the relationship between serum calcium levels during hospitalization and the incidence and prognosis of sepsis in hospitalized patients with psoriasis.

**Methods:**

A retrospective analysis of patients with concomitant psoriasis admitted for the first time was conducted, utilizing the Medical Information Mart for Intensive Care database. Machine learning techniques, along with logistic regression, Cox regression, group-based trajectory modeling (GBTM), and mediation analysis, were employed to assess the influence of serum calcium levels and other clinical indicators on the occurrence of sepsis and all-cause mortality.

**Results:**

Serum calcium exhibits a significant inverse correlation with the occurrence of sepsis [odds ratio (OR) =0.351, 95% CI: 0.265-0.463, P<0.001]. Furthermore, serum calcium levels exhibited a negative correlation with 90-day all-cause mortality [hazard ratio (HR)=0.594, 95% CI: 0.422-0.835, P=0.003] and a similar negative correlation with 365-day mortality risk (HR=0.642, 95% CI: 0.502-0.821, P<0.001). Platelet counts mediated the relationship between serum calcium and both 90-day and 365-day all-cause mortality, accounting for 24.6% and 22.0% of the mediation effect, respectively. Additionally, three distinct trajectory patterns based on serum calcium levels were identified, with the low calcium trajectory group exhibiting a higher risk of sepsis (OR=2.400, 95% CI: 1.163-5.068, P<0.001).

**Conclusion:**

Serum calcium levels serve as a significant predictive factor for the occurrence and prognosis of sepsis in hospitalized patients with psoriasis. Continuous monitoring of serum calcium levels and timely correction of hypocalcemia may contribute positively to improving patient outcomes.

## 1 Introduction

Psoriasis is a common chronic, systemic skin disease characterized by the presence of localized or widespread erythematous plaques with scales (1). The primary manifestations of psoriasis include the hyperproliferation of keratinocytes, infiltration of inflammatory cells, and abnormalities in local microvasculature (2, 3). The pathogenesis of psoriasis is complex, involving interactions among genetic factors, environmental influences, and the immune system (4). Clinically, psoriasis presents in various forms, with common types including plaque psoriasis, pustular psoriasis, erythrodermic psoriasis, and psoriatic arthritis (5). Sepsis is a systemic inflammatory response syndrome triggered by infection, with persistently high incidence and mortality rates globally, particularly in patients with chronic diseases complicated by infections, who tend to have poorer prognoses (6, 7).

Normal skin possesses an effective barrier function that prevents the invasion of external pathogens. The inflammatory response in psoriasis leads to abnormal proliferation and desquamation of the stratum corneum, exposing the skin surface to pathogens and increasing the risk of infection (8). Patients with psoriasis often experience skin lesions such as fissures and exudation, which further compromise the skin’s defensive capabilities (9). Research has shown that patients with psoriasis exhibit an imbalance in T cell subsets, particularly an increase in Th17 cells, which is closely associated with the occurrence of sepsis (10). Th17 cells promote the inflammatory response by secreting various cytokines. While they play a role in combating infections to some extent, excessive inflammatory responses can lead to the development of systemic inflammatory response syndrome (11, 12). Patients with psoriasis undergoing systemic treatments (such as immunosuppressants, biologics, small molecule agents, and corticosteroids) experience alterations in immune function. When patients with psoriasis are hospitalized for other reasons, the occurrence of sepsis is a serious adverse complication (13).

Serological markers are among the commonly used clinical indicators for disease assessment, offering the significant advantages of easy sample collection and timely result acquisition. Previous studies have utilized proteomic analysis of serum from psoriasis patients to evaluate disease severity (14). As one of the common components in serum, calcium play a crucial role in immune regulation (15). Dysregulation of serum calcium homeostasis can exacerbate inflammatory responses, and appropriate calcium levels are essential for maintaining cellular function (16). Previous studies have reported that both low and high serum calcium levels in patients with sepsis are associated with poor prognosis (17). It remains unclear whether this conclusion applies to patients with psoriasis complicated by sepsis. Furthermore, existing studies predominantly utilize cross-sectional designs, which do not elucidate the dynamic trajectory of serum calcium levels and their impact on long-term outcomes. The aim of this study is to systematically analyze the relationship between serum calcium levels and the occurrence and prognosis of sepsis in psoriasis patients, providing new insights for clinical risk stratification and intervention strategies. By analyzing longitudinal data from psoriasis patients during their initial hospitalization, this study is the first to explore the association between dynamic changes in serum calcium levels and the risk of sepsis and all-cause mortality, while also attempting to reveal the underlying mechanisms mediated by platelets.

## 2 Materials and methods

### 2.1 Study population

This study retrospectively included psoriasis patients who were hospitalized for the first time from the MIMIC-IV (Medical Information Mart for Intensive Care IV) database for clinical research. The MIMIC-IV database is a comprehensive dataset developed and maintained by the Massachusetts Institute of Technology Laboratory for Computational Physiology. It encompasses medical information related to patients admitted to the intensive care unit of Beth Israel Deaconess Medical Center (https://physionet.org/content/mimiciv/3.0/).

**Figure 1:**
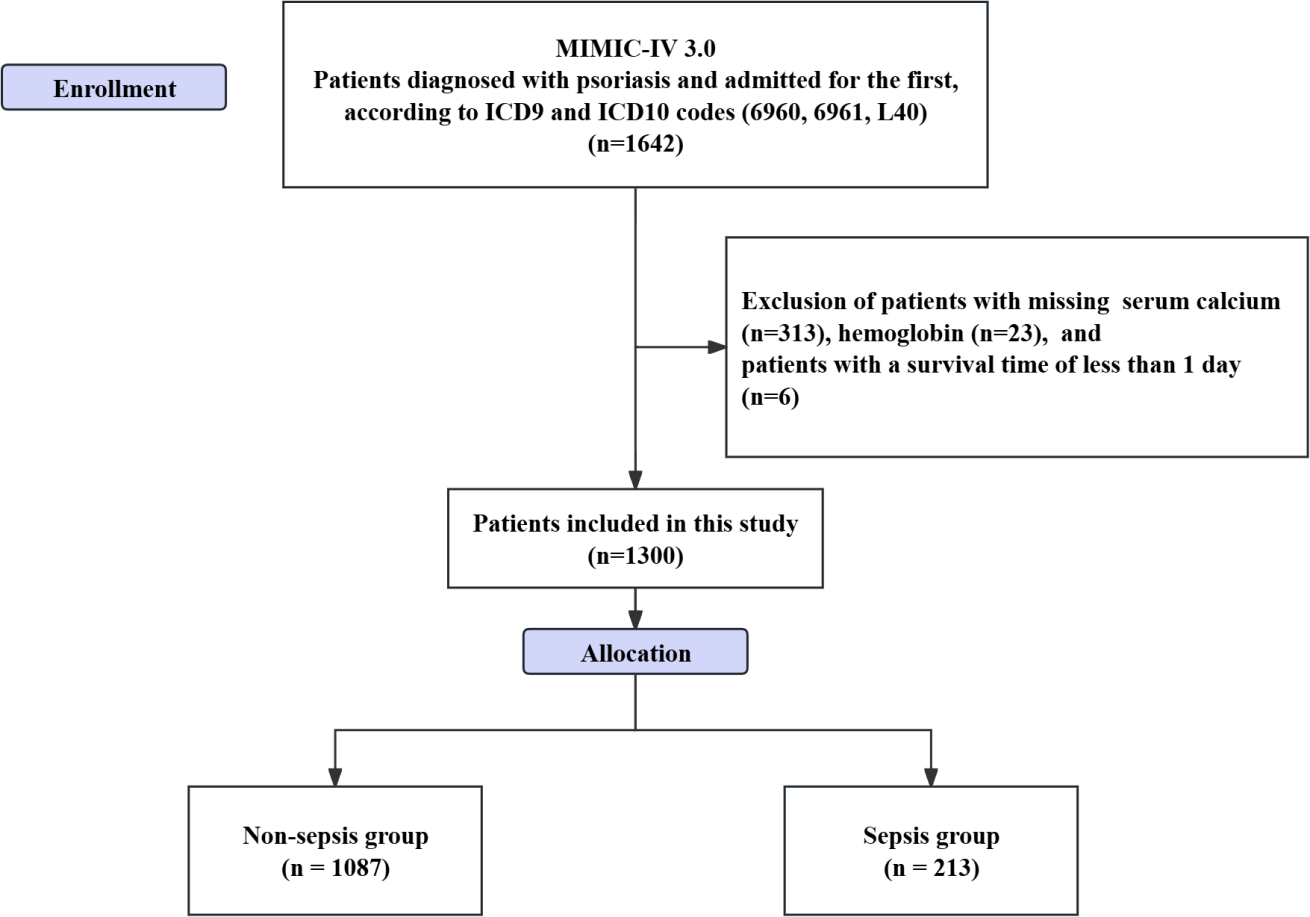
Flowchart of patient inclusion in the study.

Inclusion criteria for patients were as follows: (1) patients diagnosed with psoriasis based on ICD-9 codes (6960, 6961) and ICD-10 code (L40) in the MIMIC database; (2) patients admitted to the hospital for the first time. Exclusion criteria included: (1) patients with missing laboratory test results; and (2) patients without recorded survival time (**Figure 1**). The diagnosis of sepsis was based on the Sepsis 3.0 diagnostic criteria. Patients were required to have a concurrent infection and a Sequential Organ Failure Assessment (SOFA) score of ≥ 2 (18, 19). It is worth noting that, due to the limitations of the database, the autoimmune diseases and sepsis involved in this study were defined using ICD - 10 - CM diagnostic codes and the free - text information in patients’ discharge summaries and previous published studies were referred to (20).

Included patients were classified into sepsis and non-sepsis groups based on the occurrence of sepsis. One of the authors of this study, Zheng, successfully completed the “Data or Sample Research” training program (CITI) and passed the examination (Record ID: 39635575), subsequently obtaining access to the dataset. Considering that the MIMIC-IV database is publicly accessible and consists of de-identified patient data, this study did not require informed consent or ethical approval.

### 2.2 Data extraction

Data extraction was performed using the Decisionlinnc1.071 software (https://www.statsape.com). In this study, the clinical parameters extracted from the MIMIC database included age, sex, hematocrit, hemoglobin, platelet count, red cell distribution width, red blood cell count, white blood cell count, anion gap, serum total calcium, chloride concentration, glucose levels, potassium concentration, sodium concentration, creatinine levels, blood urea nitrogen levels, body mass index (BMI), and histories of hypertension, type 2 diabetes, coronary artery disease, chronic obstructive pulmonary disease (COPD), chronic kidney disease (CKD) and acute kidney injury (AKI). These parameters encompass hematological indices, electrolyte balance, metabolic status, and chronic disease history, aiming to provide a comprehensive assessment of the patients’ prognosis and their potential impact on the risk of sepsis. The diagnosis of sepsis was based on the Sepsis-3 criteria, identifying patients with infection and a Sequential Organ Failure Assessment (SOFA) score of ≥2 after admission (21). In the cross-sectional study section, laboratory parameters were extracted from the first biochemical analysis results obtained after patient admission. The patients’ routine blood tests were performed on the first day post - admission. The time of the first biochemical examination occurred within 7 days after admission. For the longitudinal study of serum calcium, data were collected from the first measurement taken upon admission, as well as subsequent measurements up to day seven.

### 2.3 Primary outcome and secondary outcomes

The primary outcome of this study was the incidence of sepsis in hospitalized patients with concomitant psoriasis. The secondary outcomes included the all-cause mortality rate within 90 days post-admission and the mortality rate within 365 days post-admission.

### 2.4 Establishment of the predictive model

Using the occurrence of sepsis as the outcome event, patients were randomly divided into a training set (780 cases) and a validation set (520 cases) in a 6:4 ratio (**Supplementary Table S1**). The survival time was defined as the time from admission to the occurrence of all-cause mortality, with follow-up time points set at 90 days and 365 days post-admission. A Cox proportional hazards model was established. Initially, in the training set, independent risk factors influencing the development of sepsis in patients were identified using the Least Absolute Shrinkage and Selection Operator (LASSO) regression model. Variables with non - zero coefficients in the results of LASSO regression were selected to construct the subsequent nomogram prediction model. The nomogram prediction model was constructed using logistic regression. The model’s predictive accuracy was assessed in both the training and validation sets using the Receiver Operating Characteristic (ROC) curve. The model’s discriminative ability was validated through calibration curves, and clinical decision curves were plotted to evaluate the potential benefits to patients. Furthermore, using the feature variables selected from the training set, the Shapley Additive Explanations (SHAP) values were calculated utilizing a machine learning random forest algorithm. The SHAP is a method used to explain the prediction results of machine learning models. The basic principle of this method lies in calculating the incremental impact of each feature on the model output, enabling the interpretation of the model’s behavior at both the global and local levels. The importance of the feature variables was visualized using a beeswarm plot, allowing for the further identification of clinical indicators that contribute most significantly to the occurrence of sepsis for additional analysis (22). To further refine the predictive model, we compared the predictive capabilities of five machine learning algorithms—Decision Tree, K-Nearest Neighbors (KNN), Logistic Regression, Random Forest, and XGBoost—in predicting the occurrence of sepsis within the overall sample.

### 2.5 Statistical analysis

This study utilized R software (http://www.R-project.org; version 3.4.3) for data processing. The Shapiro-Wilk test was employed to assess the normality of the data. For normally distributed continuous variables, results are presented as means ± standard deviation, and intergroup comparisons were conducted using the Student’s t-test. Categorical data were described using frequencies and percentages, with intergroup comparisons made using the Chi-square test or Fisher’s exact test. For the features selected by LASSO regression, the SHAP values were referenced to further identify the most contributive variables, leading to the construction of three distinct regression models. Model 1 served as a basic model without any adjustments for covariates. Model 2 adjusted for age and sex as covariates to eliminate potential confounding effects related to demographics. Model 3 further expanded upon Model 2 by incorporating additional features identified through LASSO regression. The regression models included both logistic regression for sepsis occurrence and Cox regression for all-cause mortality at 90 days and 365 days. Subgroup analyses were conducted to evaluate the impact of feature variables on the study outcomes across different subpopulations. Additionally, restricted cubic splines (RCS) were used to analyze the nonlinear relationships between the most contributive feature variables and the occurrence of sepsis as well as all-cause mortality. Mediation analysis was performed to assess potential mediating pathways through which feature variables influence sepsis and all-cause mortality. After establishing the impact of feature variables on sepsis and all-cause mortality in hospitalized psoriasis patients, a Group-Based Trajectory Model (GBTM) was employed to identify potential trajectories of feature variables, with sepsis occurrence as the outcome measure. The selection of the optimal number of groups involves an iterative process. A polynomial function is applied to the trajectories to determine the number of groups, with the model ranging from 1 group (indicating no significant trajectories) to 5 groups. The evaluation of model fitting criteria is considered from the following aspects: (1) Focus on evaluating the Akaike Information Criterion (AIC) and the Bayesian Information Criterion (BIC); (2) Require that each trajectory group includes at least 5% of the population; (3) The average post - hoc assignment probability (Avepp) with a value greater than 0.7; (4) The simplicity and clinical interpretability of the model (23, 24). Logistic regression was used to analyze the effects of different trajectories on the incidence of sepsis. Finally, two sensitivity analyses were conducted. The first one was to exclude potential biases that might be introduced during the data imputation process. This step involved deleting all samples with missing laboratory values or containing outliers, and then re - analyzing the impact of characteristic variables on the incidence of sepsis and all - cause mortality. The second step was to further remove patients with comorbid CKD and those using immunosuppressants based on the previous step, and then evaluate the impact of characteristic variables. In terms of missing value processing, the threshold for missing laboratory values was set at 5% (**Supplementary Table S2**), and for laboratory indicators with missing rates below 5%, multiple imputation was performed using the Predictive Mean Matching method. After imputation, the complete measurement data were assessed for potential outliers using Z-scores. Values with a Z-score greater than 3 or less than -3 were considered to be outlier (25). Subsequently, the outliers identified by the Z - score method were converted to “NA”, and the K-Nearest Neighbors (KNN) method was employed for imputation (26). A two-sided p-value of < 0.05 was considered statistically significant.

## 3 Results

### 3.1 General information of the included patients

A total of 1,300 hospitalized patients with psoriasis were included in this study, of which 213 patients (16.38%) were classified into the sepsis group, while 1,087 patients were in the non-sepsis group. In terms of biochemical parameters, the serum calcium level in the sepsis group was significantly lower than that in the non-sepsis group (8.29 ± 0.72 vs. 8.78 ± 0.60, P < 0.001). Additionally, the anion gap (14.10 ± 3.19 vs. 13.43 ± 2.98, P = 0.003), blood glucose levels (131.49 ± 43.1 vs. 119.67 ± 39.74, P < 0.001), and renal function indicators (creatinine: 1.13 ± 0.58 vs. 0.99 ± 0.47; blood urea nitrogen: 21.61 ± 11.89 vs. 17.88 ± 10.64, both P < 0.001) were significantly elevated (**Table 1**).

**Table 1:** Comparison of general characteristics between sepsis and non-sepsis groups.

Variable Names	Overall	Non-sepsis	Sepsis	P value
Number	1300	1087	213	
Age (year)	63.56 ± 14.92	63.28 ± 15.23	64.94 ± 13.19	0.138
Gender (n, %)				0.003
Female	530 (40.77)	463 (42.59)	67 (31.46)	
Male	770 (59.23)	624 (57.41)	146 (68.54)	
BMI (kg/m^2^)	28.31 ± 4.16	28.27 ± 4.25	28.47 ± 3.66	0.523
Hematocrit (n, %)	35.33 ± 6.03	35.56 ± 5.94	34.14 ± 6.33	0.002
Hemoglobin (g/dL)	11.73 ± 2.12	11.8 ± 2.1	11.36 ± 2.2	0.006
Platelet (10^9^/L)	212.26 ± 85.7	218.79 ± 84.02	178.96 ± 86.62	<0.001
RDW (%)	14.22 ± 1.63	14.13 ± 1.62	14.66 ± 1.59	<0.001
RBC (10^12^/L)	3.87 ± 0.72	3.91 ± 0.71	3.67 ± 0.74	<0.001
WBC (10^9^/L)	9.44 ± 4.51	8.96 ± 3.99	11.9 ± 5.96	<0.001
Anion Gap (mEq/L)	13.54 ± 3.02	13.43 ± 2.98	14.10 ± 3.19	0.003
Serum Calcium (mg/dL)	8.7 ± 0.65	8.78 ± 0.6	8.29 ± 0.72	<0.001
Chloride (mEq/L)	103 ± 4.53	102.91 ± 4.3	103.5 ± 5.5	0.080
GLU (mg/dL)	121.61 ± 40.53	119.67 ± 39.74	131.49 ± 43.1	<0.001
Potassium (mEq/L)	4.09 ± 0.51	4.08 ± 0.49	4.15 ± 0.58	0.049
Sodium (mEq/L)	138.64 ± 3.57	138.69 ± 3.41	138.39 ± 4.33	0.259
Creatinine (mg/dL)	1.02 ± 0.5	0.99 ± 0.47	1.13 ± 0.58	<0.001
Urea nitrogen (mg/dL)	18.49 ± 10.94	17.88 ± 10.64	21.61 ± 11.89	<0.001
Hypertension (n, %)				0.884
No	699 (53.77)	583 (53.63)	116 (54.46)	
Yes	601 (46.23)	504 (46.37)	97 (45.54)	
T2DM (n, %)				0.065
No	983 (75.62)	833 (76.63)	150 (70.42)	
Yes	317 (24.38)	254 (23.37)	63 (29.58)	
CHD (n, %)				0.007
No	966 (74.31)	824 (75.80)	142 (66.67)	
Yes	334 (25.69)	263 (24.20)	71 (33.33)	
COPD (n, %)				0.033
No	1151 (88.54)	972 (89.42)	179 (84.04)	
Yes	149 (11.46)	115 (10.58)	34 (15.96)	
AKI (n, %)				<0.001
No	1046 (80.46)	986 (90.71)	60 (28.17)	
Yes	254 (19.54)	101 (9.29)	153 (71.83)	
CKD (n, %)				0.135
No	1136 (87.38)	957 (88.04)	179 (84.04)	
Yes	164 (12.62)	130 (11.96)	34 (15.96)	
Glucocorticoid (n, %)				<0.001
No	1040 (80.00)	891 (81.97)	149 (69.95)	
Yes	260 (20.00)	196 (18.03)	64 (30.05)	
Immunosuppressant (n, %)				0.480*
No	1270 (97.69)	1060 (97.52)	210 (98.59)	
Yes	30 (2.31)	27 (2.48)	3 (1.41)	

RDW, red cell distribution width; RBC, red blood cell; WBC, white blood cell; GLU, glucose; BMI, body mass index; T2DM, type 2 diabetes mellitus; CHD, coronary heart disease; COPD, chronic obstructive pulmonary disease; AKI, acute kidney injury; CKD, chronic kidney disease. *, Compared using Fisher’s exact test.

### 3.2 Predictive model

**Figure 2:**
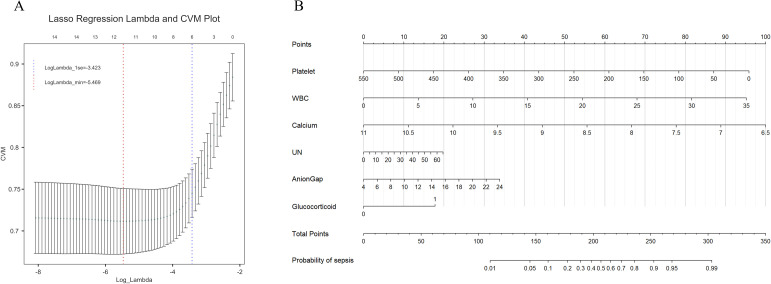
**(A)** Lasso regression for feature selection, six influencing factors were screened out, with a λ value (1se) of 0.035. The characteristic variables include platelet count, white blood cell count (WBC), serum calcium, urea nitrogen (UN), anion gap, and the use of glucocorticoids. **(B)** The constructed nomogram model based on these selected features. From the values of the six predictive factors in the chart, trace vertically upwards to intersect with the ‘Points’ line above. The scores for each predictive factor are summed to obtain a total point, and then refer to the “Total Points” axis to read the corresponding scale on the “probability of sepsis” axis below, so as to predict the risk of sepsis occurrence.

In the training set, we conducted LASSO regression analysis based on the differential indicators identified in **Table 1**. Using a lambda value of 1se = 0.035, we selected the following feature variables: platelet count, white blood cell count (WBC), calcium, blood urea nitrogen, anion gap, and glucocorticoid use (**Figure 2A**). Logical regression models were constructed using these selected characteristic variables. After the collinearity test, the variance inflation factors were all less than 10. A nomogram model was developed based on the logical regression model (**Figure 2B**).

**Figure 3:**
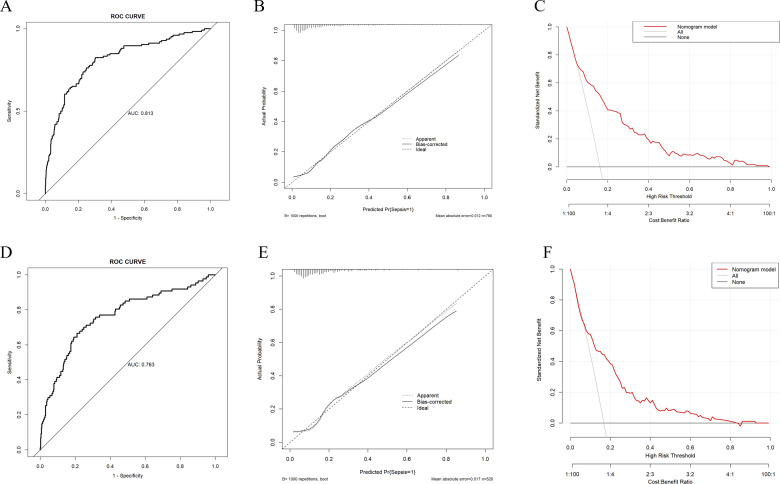
Evaluation of the predictive ability of the nomogram model **(A)** ROC curve of the training set, the AUC of nomogram was 0.813 (95%CI 0.769-0.857). **(B)** Calibration curve of the training set, “Ideal” represents the ideal reference line of the nomogram. “Apparent” represents the actual performance of the nomogram, while “Bias - corrected” represents the performance of the nomogram after correcting for bias. **(C)** Decision curve analysis (DCA) curve of the training set. **(D)** ROC curve of the validation set, the AUC of nomogram was 0.763 (95%CI 0.703-0.823). **(E)** Calibration curve of the validation set. **(F)** DCA curve of the validation set.

In the training set, the model achieved a C-index of 0.813, and the goodness-of-fit test (Hosmer-Lemeshow test) yielded a chi-squared value of 13.251 with a P-value of 0.104. The area under the curve (AUC) was 0.813, with a 95% confidence interval (CI) of 0.769-0.857 (**Figure 3A**). The calibration curve indicates a high degree of agreement between the predicted probability and the actual probability (**Figure 3B**). Moreover, the Decision Curve Analysis (DCA) also demonstrates good clinical benefit - gaining ability (**Figure 3C**). In the validation set, the C-index was 0.763, with a goodness-of-fit test yielding a chi-squared value of 8.483 and a P-value of 0.388. The AUC was 0.763, with a 95% CI of 0.703-0.823 (**Figure 3D**). Calibration curves (**Figure 3E**) and DCA curves (**Figure 3F**) also indicated that the model possesses good predictive capability in the validation sets.

**Figure 4:**
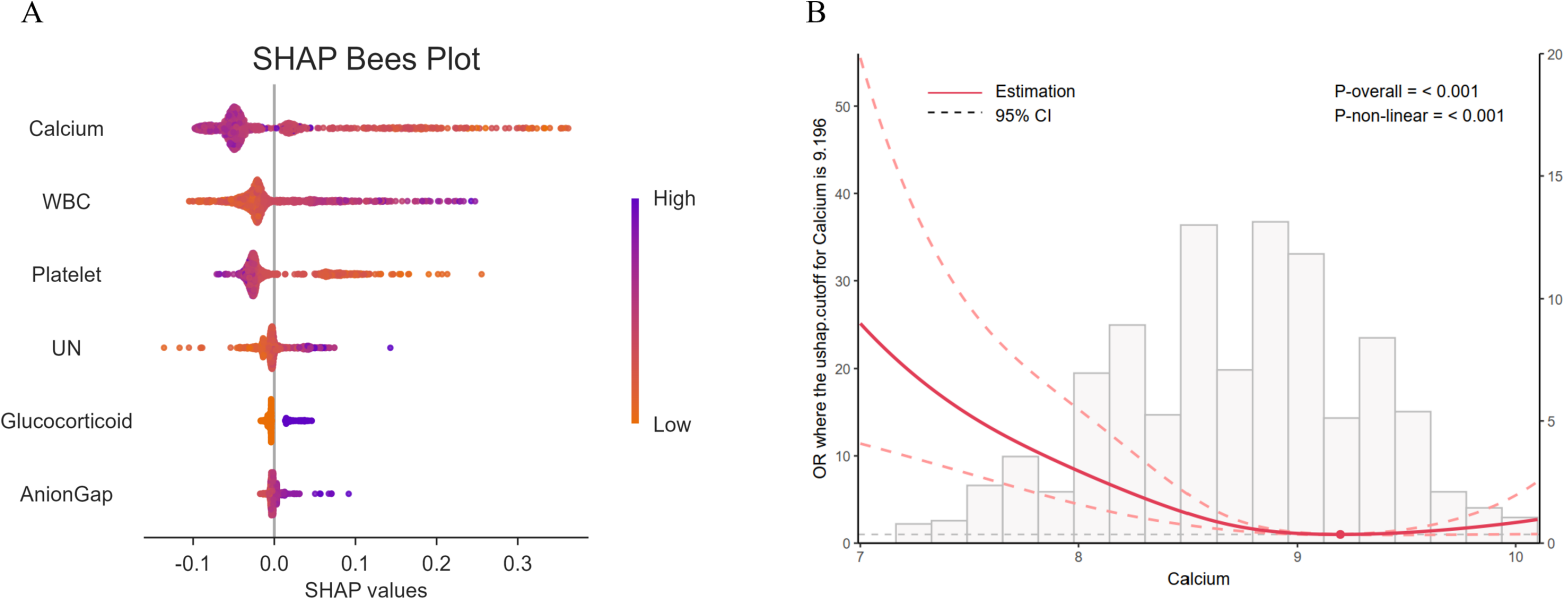
**(A)** Bee swarm plot of SHAP values calculated using the random forest algorithm. Each point represents an observation. The x - coordinate of the point is the SHAP value, and the color gradient of the point corresponds to the value of the independent variable. The arrangement of independent variables on the y - axis is consistent with the ranking of SHAP variable importance, that is, from top to bottom, the importance of independent variables decreases. **(B)** The Restricted Cubic Spline (RCS) curve analysis indicates that there is a significant non - linear relationship (P <0.001) between serum calcium and the occurrence of sepsis. UN, Urea nitrogen.

Based on the data of the training set, the SHAP values of the feature variables were calculated using the machine - learning random forest algorithm, and their importance was sorted and presented using a bee swarm plot. The results indicated that serum calcium had the greatest contribution among the selected feature variables in predicting the occurrence of sepsis (**Figure 4A**). Furthermore, the RCS curve analysis in the training set suggested a nonlinear relationship between serum calcium levels and the occurrence of sepsis (**Figure 4B**).

### 3.3 Comparison of machine learning algorithms

**Figure 5:**
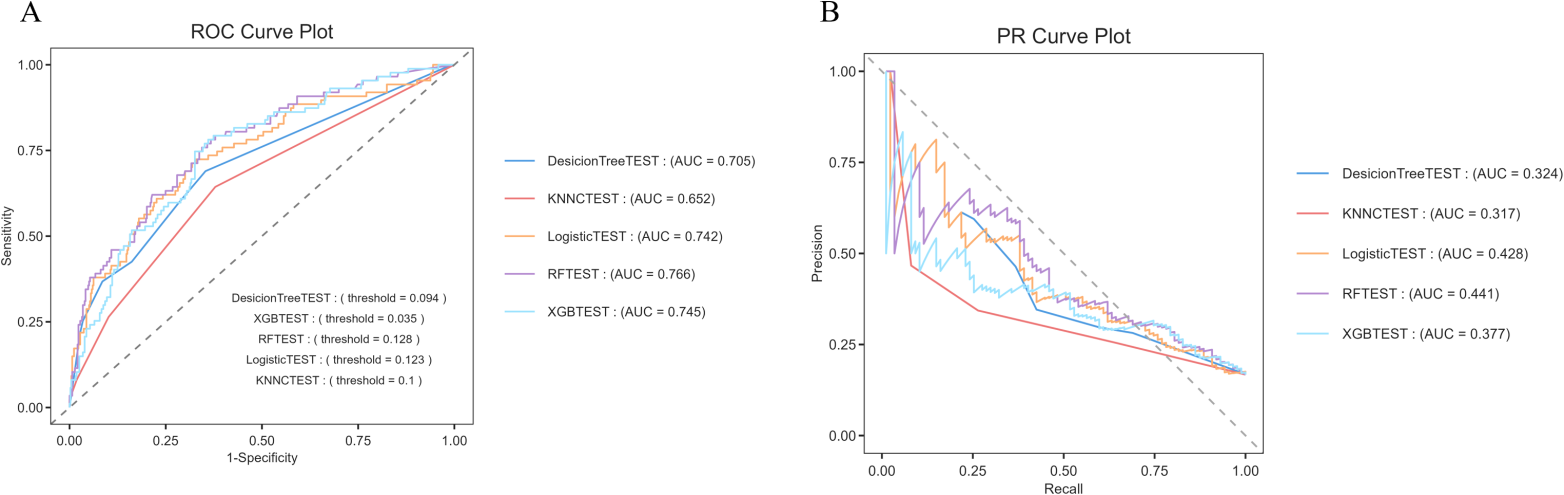
Comparison of predictive capabilities of machine learning algorithms **(A)** ROC curve, the logistic regression algorithm demonstrated the highest AUC value (0.742) in the comparison. **(B)** PR curve, the horizontal axis represents the recall rate, and the vertical axis represents the precision rate. The random forest (AUC = 0.441) and logistic regression (AUC = 0.428) had relatively better performance. Their curves were closer to the upper - right corner, indicating better performance in balancing precision and recall.

The logistic regression model demonstrated the best performance across various evaluation metrics, achieving an accuracy of 84.8%, an AUC of 0.742, a precision of 0.682, and a specificity of 0.984. In comparison, the random forest model exhibited an accuracy of 83.7%, an F1 score of 0.190, a Matthews correlation coefficient (MCC) of 0.197, and an AUC of 0.766, indicating satisfactory overall performance (**Figure 5**).

The logistic regression model emerged as the most effective tool for predicting hospitalization outcomes in patients with psoriasis, exhibiting high accuracy and excellent specificity, thus making it suitable for clinical prediction (**Supplementary Table S3**).

### 3.4 Impact of serum calcium on sepsis

We analyzed the relationship between serum calcium levels and the occurrence of sepsis during hospitalization in patients with psoriasis. The results indicated a significant negative correlation between serum calcium levels and the incidence of sepsis. In Model 1, the odds ratio (OR) for serum calcium levels was 0.290 (95% CI: 0.225–0.371, P < 0.001). In the adjusted models, the OR for serum calcium levels showed slight variation but remained significantly negatively correlated across all levels (all P < 0.05). Furthermore, after adjusting for covariates such as age, sex, platelet count, white blood cell count, blood urea nitrogen, anion gap, and glucocorticoids, the association between serum calcium levels and the occurrence of sepsis remained significant. The trend tests across all models reached statistical significance (P < 0.001) (**Table 2**).

**Table 2:** Analysis of the association between serum calcium and occurrence of sepsis.

Variable	Model 1 OR (95%CI)	P value	Model 2 OR (95%CI)	P value	Model 3 OR (95%CI)	P value
Calcium	0.290(0.225~0.371)	<0.001	0.286(0.221~0.366)	<0.001	0.351(0.265~0.463)	<0.001
Q1	Ref		Ref		Ref	
Q2	0.394(0.269~0.570)	<0.001	0.386(0.263~0.560)	0.053	0.423(0.279~0.634)	<0.001
Q3	0.180(0.111~0.282)	<0.001	0.178(0.109~0.279)	0.011	0.231(0.138~0.372)	<0.001
Q4	0.161(0.096~0.256)	<0.001	0.163(0.098~0.262)	0.004	0.220(0.127~0.368)	<0.001
P for trend		<0.001		<0.001		<0.001

Model 1: no covariates were adjusted. Model 2: age and gender were adjusted. Model 3: age, gender, platelet, white blood cell, urea nitrogen, anion gap and glucocorticoid were adjusted.

Subgroup analyses revealed that this reverse association was consistently observed across different clinical characteristics. Notably, in the subgroup analysis for AKI, a significant negative correlation between serum calcium and sepsis risk was observed in non-AKI patients (OR = 0.303, 95% CI: 0.192–0.496, P < 0.001), while this association was attenuated in AKI patients (OR = 0.595, 95% CI: 0.378–0.937, P = 0.025). Additionally, a significant interaction between the subgroups was noted (P for interaction = 0.023) (**Supplementary Table S4**).

### 3.5 Impact of serum calcium on all-cause mortality

Serum total calcium exhibited a significant negative correlation with both 90-day and 365-day all-cause mortality rates. In Model 1, which did not adjust for any covariates, the hazard ratio (HR) for total calcium associated with 90-day mortality was 0.574 (95% CI: 0.419–0.785, P < 0.001), while the HR for 365-day mortality was 0.662 (95% CI: 0.528–0.831, P < 0.001). In Model 3, which adjusted for various covariates, the HR for 90-day mortality was 0.594 (95% CI: 0.422–0.835, P = 0.003), and the HR for 365-day mortality was 0.642 (95% CI: 0.502–0.821, P < 0.001). Furthermore, analysis grouped by quartiles of total calcium revealed a significant downward trend in both 90-day and 365-day mortality rates with increasing calcium levels (P for trend = 0.012 and 0.004, respectively) (**Table 3**).

**Table 3:** Analysis of the association between serum calcium and all-cause mortality.

Outcome	Model 1 HR, 95% CI	P value	Model 2 HR, 95% CI	P value	Model 3 HR, 95% CI	P value
90-day mortality						
Calcium	0.574 (0.419 ~ 0.785)	<0.001	0.513 (0.373 ~ 0.707)	<0.001	0.594 (0.422 ~ 0.835)	0.003
Q1	Ref		Ref		Ref	
Q2	0.649 (0.379 ~ 1.112)	0.116	0.587 (0.342 ~ 1.006)	0.053	0.585 (0.334 ~ 1.025)	0.061
Q3	0.524 (0.294 ~ 0.933)	0.028	0.474 (0.265 ~ 0.845)	0.011	0.599 (0.329 ~ 1.089)	0.093
Q4	0.471 (0.258 ~ 0.860)	0.014	0.405 (0.220 ~ 0.743)	0.004	0.475 (0.250 ~ 0.901)	0.023
P for trend		0.012		0.003		0.032
365-day mortality						
Calcium	0.662 (0.528 ~ 0.831)	<0.001	0.601 (0.476 ~ 0.758)	<0.001	0.642 (0.502 ~ 0.821)	<0.001
Q1	Ref		Ref		Ref	
Q2	0.761 (0.518 ~ 1.117)	0.163	0.701 (0.478 ~ 1.030)	0.071	0.691 (0.466 ~ 1.023)	0.065
Q3	0.562 (0.369 ~ 0.855)	0.007	0.514 (0.337 ~ 0.783)	0.002	0.583 (0.379 ~ 0.898)	0.014
Q4	0.573 (0.376 ~ 0.872)	0.009	0.501 (0.328 ~ 0.765)	0.001	0.537 (0.344 ~ 0.838)	0.006
P for trend		0.004		0.001		0.005

Model 1: no covariates were adjusted. Model 2: age and gender were adjusted. Model 3: age, gender, platelet, white blood cell, urea nitrogen, anion gap and glucocorticoid were adjusted.

Subgroup analysis indicated that the serum calcium was negatively correlated with the 90 - day all - cause mortality (HR = 0.662, 95% CI: 0.476–0.920, P = 0.014). Gender-based subgroup analysis revealed that the negative correlation between serum calcium and mortality was more pronounced in male patients (HR = 0.472, 95% CI: 0.306–0.729, P < 0.001), while no significant association was observed in female patients (P = 0.870). Additionally, no significant association was found in patients with concurrent COPD (P = 0.219) (**Supplementary Table S5**). Similar effects were observed in the subgroup analysis related to 365-day all-cause mortality (**Supplementary Table S6**).

### 3.6 Mediation effect analysis

**Figure 6:**
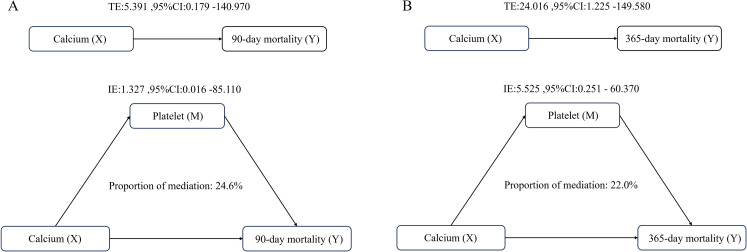
Analysis of the mediating role of platelets in the relationship between serum calcium and all-cause mortality **(A)** Analysis of 90-day all-cause mortality; **(B)** Analysis of 365-day all-cause mortality. Adjusted for age and gender.

Platelet count serves as a significant mediator in the relationship between serum calcium and 90-day all-cause mortality. The proportion of the mediation effect was found to be 0.246 (95% CI: 0.066 - 0.590, P = 0.020). In the analysis of 365-day all-cause mortality, the proportion of the mediation effect for this analysis was 0.220 (95% CI: 0.076–0.450, P < 0.001) (**Table 4**) (**Figure 6**).

**Table 4:** Platelet as a mediator variable between Calcium and the correlation of all-cause mortality.

Mediation effect	Estimate	95% CI Lower	95% CI Upper	P value
90-day mortality				
Total effect	5.391	0.179	140.970	<0.001
ACME	1.327	0.016	85.110	0.020
ADE	4.065	0.079	58.720	0.020
Proportion mediated	0.246	0.066	0.590	0.020
365-day mortality				
Total effect	24.016	1.225	149.580	<0.001
ACME	5.525	0.251	60.370	<0.001
ADE	18.491	0.969	107.080	<0.001
Proportion mediated	0.220	0.076	0.450	<0.001

ACME, average causal mediation effect, ADE, average direct effect.

### 3.7 GBTM analysis

A total of 151 patients had complete serum calcium data measured repeatedly on the 1st, 3rd, and 5th days after admission, and were finally included in the GBTM analysis. With reference to the AIC criterion, BIC criterion and taking into account the simplicity of the model, the three - trajectory model was finally determined for selection.

**Figure 7:**
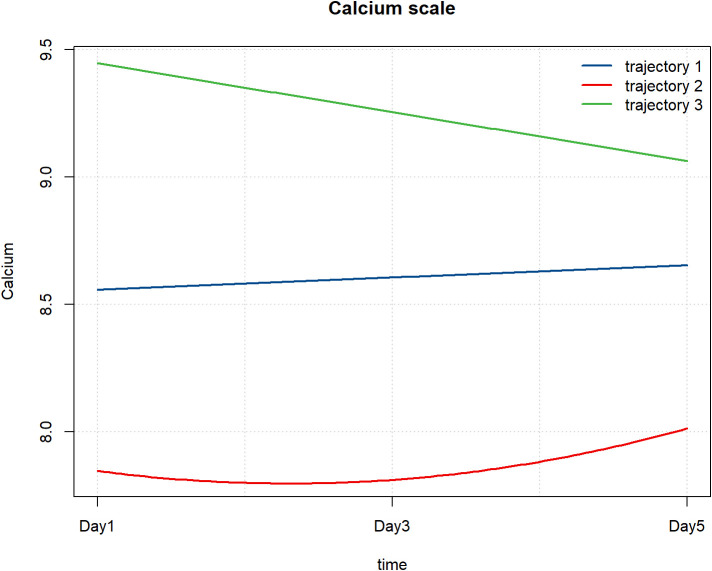
Patterns of serum calcium changes after hospital admission. Trajectory 1, slow increase trend; Trajectory 2, decreasing -increasing trend; Trajectory 3, decreasing trend.

Using a three-trajectory model, three distinct trajectory patterns were identified. Trajectory 1 exhibited a slow increase in calcium levels, ranging from 8.5 to 9. Trajectory 2 demonstrated an initial decline followed by an increase, starting below 8. Trajectory 3 began with higher calcium levels above 9 but showed a downward trend (**Figure 7**).

Comparing the differences in adverse prognostic outcomes among the three estimated populations revealed significant differences in the incidence of sepsis (P = 0.021). Specifically, Trajectory 3 had the lowest sepsis incidence at 28.57%, while Trajectory 2 exhibited the highest incidence at 55.88%. These findings may reflect variations in infection risk across the different trajectory groups (**Table 5**).

**Table 5:** Comparison of adverse outcomes among the three trajectory groups.

Outcomes	Trajectory 1 (n=62)	Trajectory 2 (n=68)	Trajectory 3 (n=21)	P value
365-day mortality (n, %)				
Survival	49 (79.03)	54 (79.41)	15 (71.43)	0.724
Dead	13 (20.97)	14 (20.59)	6 (28.57)	
90-day mortality (n, %)				
Survival	50 (80.65)	59 (86.76)	19 (90.48)	0.459
Dead	12 (19.35)	9 (13.24)	2 (9.52)	
AKI (n, %)				
No	35 (56.45)	35 (51.47)	12 (57.14)	0.817
Yes	27 (43.55)	33 (48.53)	9 (42.86)	
Sepsis (n, %)				
No	40 (64.52)	30 (44.12)	15 (71.43)	0.021
Yes	22 (35.48)	38 (55.88)	6 (28.57)	
ICU admission (n, %)				
No	26 (41.94)	18 (26.47)	10 (47.62)	0.088
Yes	36 (58.06)	50 (73.53)	11 (52.38)	

AKI, acute kidney injury; ICU, intensive care unit.

### 3.8 Logistic regression analysis of different trajectory groups

In Model 1, the risk of sepsis for patients in Trajectory 2 was significantly increased compared to the baseline group (Trajectory 1), with an OR of 2.303 (95% CI: 1.144-4.723, P = 0.021). This indicates that patients in Trajectory 2 have a higher risk of developing sepsis. After adjusting for age and sex in Model 2, this association remained significant, with an OR of 2.400 (95% CI: 1.163-5.068, P = 0.019). This further supports the conclusion that patients in Trajectory 2 are at a higher risk for sepsis (**Table 6**).

**Table 6:** Logistic regression analysis of trajectory and risk of sepsis occurrence.

Trajectory	Model 1 OR (95%CI)	P value	Model 2 OR (95%CI)	P value
Trajectory 1	Ref		Ref	
Trajectory 2	2.303(1.144-4.723)	0.021	2.400(1.163-5.068)	0.019
Trajectory 3	0.727(0.231-2.073)	0.563	0.714(0.225-2.060)	0.546

Model 1: no covariates were adjusted. Model 2: age and gender were adjusted.

### 3.9 Sensitivity analysis

In the sensitivity analysis of the retained sample, comparisons between the sepsis group and the non-sepsis group indicated consistent inter-group difference metrics with those observed post-imputation (**Supplementary Table S7**). Serum calcium levels consistently showed a significant inverse correlation with the occurrence of sepsis (**Supplementary Table S8**). Regarding the impact on all-cause mortality at 90 and 365 days, significant inverse association was observed across all three models (**Supplementary Table S9**). The RCS curve analysis also revealed a non-linear relationship between calcium levels and the incidence of sepsis, as well as all-cause mortality at 365 days (**Supplementary Figure S1**). Additionally, the mediation analysis further supports the mediating effect of platelets (**Supplementary Table S10**). After further excluding patients with comorbid CKD and those using immunosuppressants, the impact of serum calcium on the incidence of sepsis (**Supplementary Table S11**), the impact of serum calcium on all - cause mortality (**Supplementary Table S12**), and the results of the mediation analysis (**Supplementary Table S13**) were consistent with the previous findings.

## 4 Discussion

This study delves into the potential mechanisms by which serum calcium levels may influence the occurrence of sepsis in patients with psoriasis, revealing a significant reverse correlation between serum calcium levels and both the incidence and prognosis of sepsis (OR = 0.351, 95% CI: 0.265-0.463, P < 0.001). By analyzing hospitalization data from psoriasis patients in the MIMIC-IV database, we found that serum calcium is also negatively correlated with all-cause mortality at 90 days (HR = 0.594, 95% CI: 0.422-0.835, P = 0.003) and at 365 days (HR = 0.642, 95% CI: 0.502-0.821, P < 0.001). The findings suggest that serum calcium may influence the susceptibility of psoriasis patients to sepsis and their prognosis through their critical role in immune regulation. Additionally, we observed a mediating effect of platelets in the relationship between serum calcium and both sepsis and mortality rates, further confirming the compounded effects of calcium homeostasis imbalance in the context of chronic inflammation. This highlights the importance of maintaining calcium levels for improving outcomes in this patient population.

Calcium plays a crucial role in various physiological processes, with their concentration changes both intracellularly and extracellularly having significant effects. A study on calcium ion levels and survival rates in intensive care unit patients found that lower calcium ion levels significantly increased the risk of mortality. Additionally, the time-weighted average mean arterial pressure (TWA-MAP-0) and blood pressure reactivity parameters (TWA-MAP-0/NE) served as partial mediators, explaining 4.55% and 2.6% of the total effect, respectively (27). This study suggests that calcium ions may influence vascular tone and hemodynamic status, thereby regulating blood pressure and ultimately affecting patient survival rates. Recent studies have indicated that calcium are also pivotal in the pathogenesis and progression of psoriasis (28). Calcium are involved in cellular signal transduction, as well as in cell proliferation and differentiation. They also play a significant role in immune responses and inflammatory processes (29). Elevated calcium levels promote the differentiation of keratinocytes and enhance their barrier function (30, 31). In psoriasis patients, calcium signaling pathways may be disrupted, leading to abnormal proliferation and dysfunction of keratinocytes, which can further exacerbate skin lesions. This dysregulation contributes to the characteristic features of psoriasis, including increased cell turnover and impaired barrier function (32). The immune system of psoriasis patients is somewhat activated, and secondary infections may trigger a more pronounced systemic inflammatory response. J. Knuever et al. observed in a case report that severe hypocalcemia may have triggered an eruption of pustular psoriasis in a patient newly diagnosed with primary hypoparathyroidism. Following calcium infusion, the patient’s psoriatic plaques rapidly improved without the need for additional treatment (33). Chronic hypocalcemia promotes an inflammatory microenvironment by activating the TRPV6 calcium channel, which enhances the differentiation of Th17 cells and the secretion of IL-17A (34). A study that also focused on serum calcium levels found an inverse correlation between serum calcium and the 28 - day mortality risk in septic patients (HR = 0.82, 95%CI: 0.76 - 0.88). When the serum calcium level was below 9.0 mg/dL, for every 1.0 mg/dL increase in serum calcium, the 28 - day mortality risk decreased by 58% (HR, 0.42; 95% CI, 0.37 - 0.48) (35). The potential mechanism underlying the correlation between low serum calcium levels and sepsis may be that the inflammatory state of sepsis causes the flow of calcium ions from outside the cell into the cell (36, 37). This study conducted a comprehensive assessment with a large sample size, revealing a significant association between low serum calcium levels and an elevated risk of developing sepsis. This finding further underscores the importance of serum calcium in the immune status of patients with psoriasis.

This study utilized hospitalization data from patients with psoriasis within the MIMIC-IV database to develop a clinical prediction model for assessing the risk of sepsis in this population. The selected variables platelet count, white blood cell count, serum calcium levels, blood urea nitrogen, anion gap, and glucocorticoid use were all closely related to the pathophysiological processes of sepsis. Notably, the reverse association of serum calcium levels remained significant after multivariable adjustment, indicating its independent predictive value. Additionally, the association between thrombocytopenia and sepsis may be linked to abnormalities in coagulation function and the release of inflammatory mediators (38, 39). Elevated white blood cell counts indicate the activation of systemic inflammatory responses (40). These variable selections not only validate the effectiveness of previously identified risk factors for sepsis but also incorporate serum calcium into the sepsis prediction model for psoriasis patients for the first time, providing a new biomarker for clinical monitoring. SHAP value analysis further reveals the contribution of feature variables, showing that serum calcium has the highest SHAP value in predicting the occurrence of sepsis, significantly surpassing other indicators. This finding suggests that serum calcium levels are a core factor influencing the sepsis risk in psoriasis patients.

This study identified three dynamic patterns of serum calcium levels in hospitalized patients with psoriasis through GBTM analysis. The incidence of sepsis in trajectory group 2 was significantly higher than in trajectory group 1, suggesting that a sustained low calcium state may exceed the body’s compensatory threshold, reflecting the severity of inflammation. This research is the first to introduce trajectory analysis into the study of psoriasis complications, providing a theoretical basis for dynamic monitoring. It is recommended that hospitalized psoriasis patients undergo continuous serum calcium monitoring for at least three days, and that calcium supplementation be initiated for those in trajectory group 2, with target levels maintained between 8.5-9.5 mg/dL. In patients with psoriasis, disturbances in vitamin D metabolism represent a significant mechanism. Vitamin D deficiency is prevalent among individuals with psoriasis. Extensive skin lesions may impair the synthesis and activation of vitamin D, leading to reduced intestinal calcium absorption. Additionally, the thickened stratum corneum allows only one-third of the ultraviolet B radiation to penetrate compared to healthy skin, resulting in a multifactorial reduction in vitamin D synthesis (41). The deficiency of active vitamin D diminishes intestinal calcium absorption efficiency, further exacerbating hypocalcemia. On the other hand, vitamin D must be converted to its active form (1,25-dihydroxyvitamin D) in the kidneys; if renal function is compromised, this process may be adversely affected, leading to decreased vitamin D levels (42). Taken together, these pathological mechanisms suggest that the calcium ion levels in patients on trajectory 2 reflect a slow increase from a low baseline, indirectly indicating the severity of the condition.

Calcium, as an important second messenger, may play a crucial role in the pathogenesis of psoriasis by regulating signaling pathways such as calcium/calmodulin-dependent protein kinase IV (CaMK4) and calcium/calmodulin-dependent protein kinase II-γ (CaMK2γ). Studies have shown that the expression of CaMK4 is significantly increased in the skin lesions of psoriasis patients. This upregulation inhibits macrophages from producing the anti-inflammatory factor IL-10 while promoting the secretion of the pro-inflammatory factor IL-17, thereby exacerbating the inflammatory response (30). CaMK2γ is primarily expressed in the sympathetic nerves of the skin, and its activity is activated by stress factors in psoriasis. CaMK2γ promotes the secretion of norepinephrine by upregulating tyrosine hydroxylase. NE then acts on γδT cells and enhances the production of IL-17 through the ARβ1–NF-κB–p38 axis, thereby exacerbating the inflammatory phenotype of psoriasis. This pathway highlights the interplay between the nervous system and immune responses in the context of psoriasis, suggesting potential therapeutic targets for modulating these interactions (43).

The mediation effect model indicates that platelets mediate the impact of serum calcium on short-term mortality in patients. A low-calcium environment activates TRPC6 channels, leading to the release of pro-inflammatory mediators such as platelet factor 4 (PF4) and β-thromboglobulin from platelets (44). Platelet aggregation can further exacerbate microcirculatory disturbances (45). Abnormal calcium levels not only participate directly in the pathological processes of psoriasis but may also indirectly affect overall survival rates by influencing comorbidities such as cardiovascular diseases. In subgroup analyses, this study observed a significant negative correlation between serum calcium levels and 90-day all-cause mortality in male patients (HR=0.472, 95% CI: 0.306-0.729, P<0.001), while no similar association was found in female patients (P=0.870). Furthermore, in patients with COPD, the relationship between calcium levels and mortality did not reach statistical significance (P=0.219), and an interaction between these factors was noted. These findings may be attributed to hormonal differences and the complex pathophysiological state associated with COPD and its complications, which may obscure the independent effects of serum calcium. This underscores the importance of considering the interactions among various variables when evaluating prognostic factors in patients with chronic diseases. In managing psoriasis patients, it is crucial to emphasize individualized differences, particularly the impact of gender and COPD comorbidities on the relationship between calcium levels and clinical outcomes.

This study has several limitations. First, the data were sourced from a single database, which may introduce selection bias and is constrained by the limitations of clinical parameter extraction from the database. Important treatment-related variables, such as the use of immunosuppressants, were not included and require further validation in prospective studies. Meanwhile, trajectory models usually require a sufficient sample size for modeling. This study is limited by the relatively small sample size included in the trajectory model analysis, and can only provide some observational phenomena. It awaits verification with a larger sample size by combining multi - center samples. Second, the predictive performance of the model declined for certain subgroups, indicating the need for optimization tailored to specific populations. Thirdly, as an observational study, this research is insufficient to determine whether hypocalcemia is the cause or the result of systemic immune dysfunction in patients with psoriasis complicated by sepsis. Future research should incorporate multicenter cohort studies to explore more comprehensive predictive indicators and develop dynamic monitoring tools for the precise prevention and control of sepsis in psoriasis patients.

## 5 Conclusion

Serum calcium levels have a significant reverse association with the occurrence of sepsis in hospitalized patients with psoriasis, with lower calcium levels associated with a higher risk of sepsis. Additionally, serum calcium levels influence the 90-day and 365-day all-cause mortality rates following hospital admission in these patients. Mediation analysis suggests that calcium may impact disease progression through their effect on platelet levels. Clinically, dynamic monitoring of serum calcium levels in hospitalized patients with psoriasis, along with timely correction of any deficiencies, may positively influence patient outcomes.

## Data Availability

The original contributions presented in the study are included in the article/**Supplementary Material**. Further inquiries can be directed to the corresponding author/s.

## Supplementary material

Supplementary Figure 1 The RCS curve analysis also revealed a non-linear relationship between calcium levels and the incidence of sepsis, as well as all-cause mortality at 365 days.
