# Supplementary figures and images for: Impact of serum calcium levels on the occurrence of sepsis and prognosis in hospitalized patients with concomitant psoriasis: a retrospective study based on the MIMIC-IV database
Source: Front Immunol. 2025 Jul 22;16:1621231. doi: 10.3389/fimmu.2025.1621231 (PMC12322502; doi:10.3389/fimmu.2025.1621231)

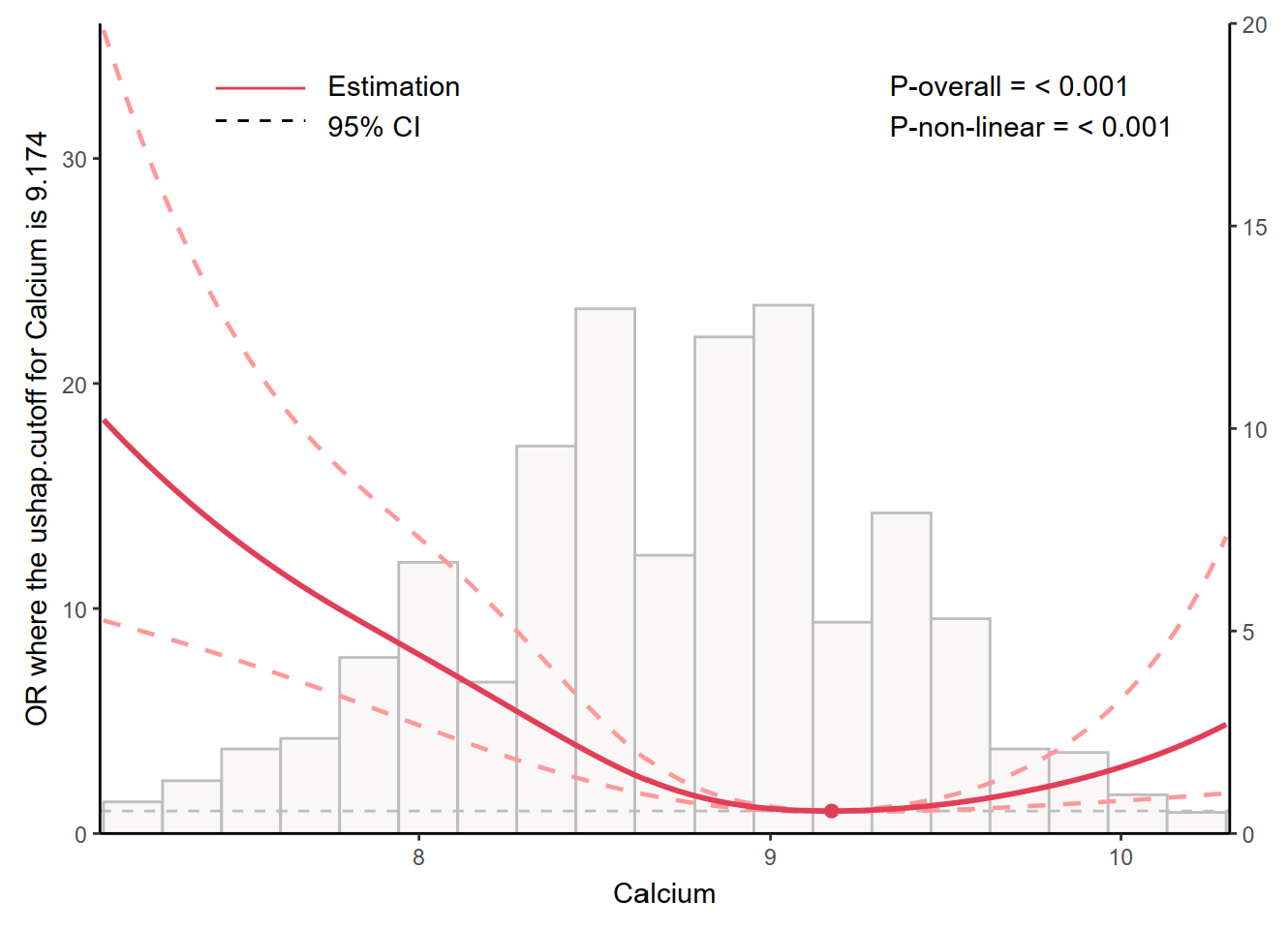

Supplement: Supplementary Figure 1 — The RCS curve analysis also revealed a non-linear relationship between calcium levels and the incidence of sepsis, as well as all-cause mortality at 365 days. [file Image1.tiff]
