# Supplementary material for: Impact of serum calcium levels on the occurrence of sepsis and prognosis in hospitalized patients with concomitant psoriasis: a retrospective study based on the MIMIC-IV database
Source: Front Immunol. 2025 Jul 22;16:1621231. doi: 10.3389/fimmu.2025.1621231 (PMC12322502; doi:10.3389/fimmu.2025.1621231)
Supplement: Supplementary file 2 [file Table1.docx]

| Table S1 Comparison of components between training and validation sets | | | | |
| --- | --- | --- | --- | --- |
| Variable Names | Overall | Training set | Validation set | P value |
| Number | 1300 | 780 | 520 |  |
| Age (year) | 63.56±14.92 | 63.74±14.68 | 63.28±15.29 | 0.583 |
| Gender (%) |  |  |  |  |
| Female | 530 (40.77) | 316 (40.51) | 214 (41.15) | 0.863 |
| Male | 770 (59.23) | 464 (59.49) | 306 (58.85) |  |
| BMI (kg/m^2^) | 28.31±4.16 | 28.21±4.17 | 28.45±4.14 | 0.322 |
| Hematocrit (n, %) | 35.33±6.03 | 35.28±6.07 | 35.41±5.97 | 0.699 |
| Hemoglobin (g/dL) | 11.73±2.12 | 11.71±2.13 | 11.76±2.11 | 0.691 |
| Platelet (10^9^/L) | 212.26±85.7 | 210.2±85.43 | 215.36±86.08 | 0.288 |
| RDW (%) | 14.22±1.63 | 14.15±1.52 | 14.31±1.77 | 0.083 |
| RBC (10^12^/L) | 3.87±0.72 | 3.86±0.72 | 3.89±0.72 | 0.401 |
| WBC (10^9^/L) | 9.44±4.51 | 9.58±4.61 | 9.25±4.34 | 0.200 |
| Anion Gap (mEq/L) | 13.54±3.02 | 13.49±3.04 | 13.63±3 | 0.403 |
| Calcium Total (mg/dL) | 8.7±0.65 | 8.7±0.64 | 8.7±0.65 | 0.831 |
| Chloride (mEq/L) | 103±4.53 | 103.26±4.45 | 102.61±4.61 | 0.011 |
| GLU (mg/dL) | 121.61±40.53 | 121.05±39.81 | 122.45±41.62 | 0.541 |
| Potassium (mEq/L) | 4.09±0.51 | 4.09±0.5 | 4.09±0.53 | 0.825 |
| Sodium (mEq/L) | 138.64±3.57 | 138.69±3.62 | 138.57±3.51 | 0.546 |
| Creatinine (mg/dL) | 1.02±0.5 | 1.03±0.51 | 1±0.47 | 0.277 |
| Urea nitrogen (mg/dL) | 18.49±10.94 | 18.71±10.95 | 18.17±10.93 | 0.391 |
| Hypertension (n, %) |  |  |  |  |
| No | 699 (53.77) | 413 (52.95) | 286 (55.00) | 0.503 |
| Yes | 601 (46.23) | 367 (47.05) | 234 (45.00) |  |
| T2DM (n, %) |  |  |  |  |
| No | 983 (75.62) | 597 (76.54) | 386 (74.23) | 0.377 |
| Yes | 317 (24.38) | 183 (23.46) | 134 (25.77) |  |
| CHD (n, %) |  |  |  |  |
| No | 966 (74.31) | 584 (74.87) | 382 (73.46) | 0.613 |
| Yes | 334 (25.69) | 196 (25.13) | 138 (26.54) |  |
| COPD (n, %) |  |  |  |  |
| No | 1151 (88.54) | 690 (88.46) | 461 (88.65) | 0.986 |
| Yes | 149 (11.46) | 90 (11.54) | 59 (11.35) |  |
| AKI (n, %) |  |  |  |  |
| No | 1046 (80.46) | 633 (81.15) | 413 (79.42) | 0.484 |
| Yes | 254 (19.54) | 147 (18.85) | 107 (20.58) |  |
| Sepsis (%) |  |  |  |  |
| No | 1087 (83.62) | 654 (83.85) | 433 (83.27) | 0.842 |
| Yes | 213 (16.38) | 126 (16.15) | 87 (16.73) |  |
| Glucocorticoid (n, %) |  |  |  | 0.436 |
| No | 1040 (80.00) | 630 (80.77) | 410 (78.85) |  |
| Yes | 260 (20.00) | 150 (19.23) | 110 (21.15) |  |
| Immunosuppressant (n, %) |  |  |  | 0.187 |
| No | 1270 (97.69) | 766 (98.21) | 504 (96.92) |  |
| Yes | 30 (2.31) | 14 (1.79) | 16 (3.08) |  |
| RDW, red cell distribution width; RBC, red blood cell; WBC, white blood cell; GLU, glucose; BMI, body mass index; T2DM, type 2 diabetes mellitus; CHD, coronary heart disease; COPD, chronic obstructive pulmonary disease; AKI, acute kidney injury. *, Compared using Fisher's exact test. | | | | |

| Table S2 Comparison of baseline characteristics before data imputation | | | | |
| --- | --- | --- | --- | --- |
| Variable Names | Missing percentage (%) | Non sepsis group | Sepsis group | P value |
| N |  | 1087 | 213 |  |
| Age (year) | 0.0 | 63.28±15.23 | 64.94±13.19 | 0.14 |
| Gender (n, %) |  |  |  | <0.01 |
| Female | 0.0 | 463 (42.59) | 67 (31.46) |  |
| Male |  | 624 (57.41) | 146 (68.54) |  |
| BMI (kg/m^2^) | 0.0 | 28.24±4.35 | 28.47±3.66 | 0.46 |
| Hematocrit (n, %) | 0.0 | 35.56±5.94 | 34.11±6.63 | <0.01 |
| Hemoglobin (g/dL) | 0.0 | 11.8±2.1 | 11.33±2.25 | <0.01 |
| Platelet (10^9^/L) | 0.4 | 223.94±95.66 | 187.71±107.32 | <0.01 |
| RDW (%) | 0.2 | 14.26±2.01 | 14.97±2.28 | <0.01 |
| RBC (10^12^/L) | 0.2 | 3.91±0.71 | 3.67±0.78 | <0.01 |
| WBC (10^9^/L) | 0.3 | 9.24±6.93 | 12.82±7.91 | <0.01 |
| Anion Gap (mEq/L) | 0.0 | 13.46±3.06 | 14.47±3.9 | <0.01 |
| Calcium Total (mg/dL) | 0.0 | 8.77±0.69 | 8.24±0.87 | <0.01 |
| Chloride (mEq/L) | 0.0 | 102.86±4.53 | 103.49±6.32 | 0.08 |
| GLU (mg/dL) | 0.0 | 123.28±50.79 | 142.09±66.42 | <0.01 |
| Potassium (mEq/L) | 0.2 | 4.09±0.51 | 4.22±0.69 | <0.01 |
| Sodium (mEq/L) | 0.0 | 138.6±3.66 | 138.18±4.76 | 0.15 |
| Creatinine (mg/dL) | 0.0 | 1.07±0.87 | 1.3±1.02 | <0.01 |
| Urea nitrogen (mg/dL) | 0.2 | 18.62±12.94 | 24.1±19.06 | <0.01 |
| Hypertension (n, %) | 0.0 |  |  |  |
| No |  | 583 (53.63) | 116 (54.46) | 0.88 |
| Yes |  | 504 (46.37) | 97 (45.54) |  |
| T2DM (n, %) | 0.0 |  |  |  |
| No |  | 833 (76.63) | 150 (70.42) | 0.07 |
| Yes |  | 254 (23.37) | 63 (29.58) |  |
| CHD (n, %) | 0.0 |  |  |  |
| No |  | 824 (75.80) | 142 (66.67) | 0.01 |
| Yes |  | 263 (24.20) | 71 (33.33) |  |
| COPD (n, %) | 0.0 |  |  |  |
| No |  | 972 (89.42) | 179 (84.04) | 0.03 |
| Yes |  | 115 (10.58) | 34 (15.96) |  |
| AKI (n, %) | 0.0 | 988 (90.64) | 61 (28.24) | <0.01 |
| No |  | 102 (9.36) | 155 (71.76) |  |
| Yes |  |  |  |  |
| Glucocorticoid (n, %) | 0 |  |  | <0.001 |
| No |  | 891 (81.97) | 149 (69.95) |  |
| Yes |  | 196 (18.03) | 64 (30.05) |  |
| Immunosuppressant (n, %) | 0 |  |  | 0.480* |
| No |  | 1060 (97.52) | 210 (98.59) |  |
| Yes |  | 27 (2.48) | 3 (1.41) |  |
| RDW, red cell distribution width; RBC, red blood cell; WBC, white blood cell; GLU, glucose; BMI, body mass index; T2DM, type 2 diabetes mellitus; CHD, coronary heart disease; COPD, chronic obstructive pulmonary disease; AKI, acute kidney injury. *, Compared using Fisher's exact test. | | | | |

| Table S3 Comparison of different machine learning predictive parameters | | | | | | | | |
| --- | --- | --- | --- | --- | --- | --- | --- | --- |
| Model Name | Accuracy | F1-Score | MCC | AUC | Presicion | Specificity | FNR | FPR |
| KNNC | 0.831 | 0.137 | 0.138 | 0.652 | 0.467 | 0.982 | 0.920 | 0.018 |
| Logistic | 0.848 | 0.275 | 0.290 | 0.742 | 0.682 | 0.984 | 0.828 | 0.016 |
| RF | 0.837 | 0.190 | 0.197 | 0.766 | 0.556 | 0.982 | 0.885 | 0.018 |
| XGBoost | 0.810 | 0.308 | 0.210 | 0.745 | 0.393 | 0.921 | 0.747 | 0.079 |
| Decision Tree | 0.846 | 0.355 | 0.317 | 0.705 | 0.595 | 0.965 | 0.747 | 0.035 |
| KNNC, k-nearest neighbors classifier; MCC, matthews correlation coefficient; AUC, area under the curve; FNR, false negative rate; FPR, false positive rate; RF, random forest. | | | | | | | | |

| Table S4 Subgroup analysis of the impact of calcium on the development of sepsis | | | | |
| --- | --- | --- | --- | --- |
| Variables | n (%) | OR (95%CI) | *P* | P for interaction |
|  |  |  |  |  |
| All patients | 1300 (100.00) | 0.360 (0.274 ~ 0.474) | <0.001 |  |
| Gender |  |  |  | 0.233 |
| F | 530 (40.77) | 0.425 (0.262 ~ 0.690) | 0.001 |  |
| M | 770 (59.23) | 0.321 (0.227 ~ 0.453) | <0.001 |  |
| Hypertension |  |  |  | 0.377 |
| No | 699 (53.77) | 0.391 (0.269 ~ 0.570) | <0.001 |  |
| Yes | 601 (46.23) | 0.317 (0.210 ~ 0.478) | <0.001 |  |
| T2DM |  |  |  | 0.586 |
| No | 983 (75.62) | 0.380 (0.275 ~ 0.525) | <0.001 |  |
| Yes | 317 (24.38) | 0.285 (0.164 ~ 0.493) | <0.001 |  |
| CHD |  |  |  | 0.058 |
| No | 966 (74.31) | 0.290 (0.205 ~ 0.411) | <0.001 |  |
| Yes | 334 (25.69) | 0.503 (0.311 ~ 0.814) | 0.005 |  |
| COPD |  |  |  | 0.412 |
| No | 1151 (88.54) | 0.376 (0.279 ~ 0.507) | <0.001 |  |
| Yes | 149 (11.46) | 0.225 (0.101 ~ 0.501) | <0.001 |  |
| AKI |  |  |  | 0.023 |
| No | 1046 (80.46) | 0.303 (0.192 ~ 0.496) | <0.001 |  |
| Yes | 254 (19.54) | 0.595 (0.378 ~ 0.937) | 0.025 |  |
| Age |  |  |  | 0.210 |
| <65 | 657 (50.54) | 0.326 (0.218 ~ 0.488) | <0.001 |  |
| ≥65 | 643 (49.46) | 0.376 (0.254 ~ 0.556) | <0.001 |  |
| OR: Odds Ratio, CI: Confidence Interval. T2DM, type 2 diabetes mellitus; CHD, coronary heart disease; COPD, chronic obstructive pulmonary disease; AKI, acute kidney injury. | | | | |

| Table S5 Subgroup analysis of the impact of calcium on 90-day all-cause mortality | | | | |
| --- | --- | --- | --- | --- |
| Variables | n (%) | HR (95%CI) | *P* | P for interaction |
|  |  |  |  |  |
| All patients | 1300 (100.00) | 0.662 (0.476 ~ 0.920) | 0.014 |  |
| Gender |  |  |  | 0.069 |
| Female | 530 (40.77) | 1.023 (0.617 ~ 1.769) | 0.870 |  |
| Male | 770 (59.23) | 0.472 (0.306 ~ 0.729) | <0.001 |  |
| Hypertension |  |  |  | 0.408 |
| No | 699 (53.77) | 0.753 (0.475 ~ 1.196) | 0.229 |  |
| Yes | 601 (46.23) | 0.583 (0.364 ~ 0.935) | 0.025 |  |
| T2DM |  |  |  | 0.515 |
| No | 983 (75.62) | 0.623 (0.422 ~ 0.919) | 0.017 |  |
| Yes | 317 (24.38) | 0.796 (0.424 ~ 1.496) | 0.479 |  |
| CHD |  |  |  | 0.628 |
| No | 966 (74.31) | 0.724 (0.492 ~ 1.066) | 0.102 |  |
| Yes | 334 (25.69) | 0.521 (0.286 ~ 0.963) | 0.038 |  |
| COPD |  |  |  | 0.018 |
| No | 1151 (88.54) | 0.538 (0.371 ~ 0.782) | 0.001 |  |
| Yes | 149 (11.46) | 1.549 (0.771 ~ 3.112) | 0.219 |  |
| AKI |  |  |  | 0.166 |
| No | 1046 (80.46) | 0.969 (0.600 ~ 1.565) | 0.897 |  |
| Yes | 254 (19.54) | 0.596 (0.370 ~ 0.960) | 0.033 |  |
| Age |  |  |  | 0.585 |
| <65 | 657 (50.54) | 0.676 (0.350 ~ 1.306) | 0.244 |  |
| ≥65 | 643 (49.46) | 0.598 (0.401 ~ 0.891) | 0.011 |  |
| HR: Hazard Ratio, CI: Confidence Interval. T2DM, type 2 diabetes mellitus; CHD, coronary heart disease; COPD, chronic obstructive pulmonary disease; AKI, acute kidney injury. | | | | |

| Table S6 Subgroup analysis of the impact of calcium on 365-day all-cause mortality | | | | |
| --- | --- | --- | --- | --- |
| Variables | n (%) | HR (95%CI) | *P* | P for interaction |
|  |  |  |  |  |
| All patients | 1300 (100.00) | 0.715 (0.564 ~ 0.906) | 0.005 |  |
| Gender |  |  |  | 0.008 |
| Female | 530 (40.77) | 1.107 (0.757 ~ 1.619) | 0.600 |  |
| Male | 770 (59.23) | 0.532 (0.392 ~ 0.722) | <0.001 |  |
| Hypertension |  |  |  | 0.015 |
| No | 699 (53.77) | 0.928 (0.669 ~ 1.289) | 0.657 |  |
| Yes | 601 (46.23) | 0.537 (0.380 ~ 0.760) | <0.001 |  |
| T2DM |  |  |  | 0.201 |
| No | 983 (75.62) | 0.638 (0.481 ~ 0.847) | 0.002 |  |
| Yes | 317 (24.38) | 0.954 (0.613 ~ 1.487) | 0.611 |  |
| CHD |  |  |  | 0.656 |
| No | 966 (74.31) | 0.760 (0.572 ~ 1.011) | 0.059 |  |
| Yes | 334 (25.69) | 0.583 (0.370 ~ 0.919) | 0.020 |  |
| COPD |  |  |  | 0.048 |
| No | 1151 (88.54) | 0.649 (0.498 ~ 0.846) | 0.001 |  |
| Yes | 149 (11.46) | 1.039 (0.599 ~ 1.804) | 0.892 |  |
| AKI |  |  |  | 0.573 |
| No | 1046 (80.46) | 0.836(0.615 ~ 1.136) | 0.232 |  |
| Yes | 254 (19.54) | 0.655 (0.443 ~ 0.970) | 0.035 |  |
| Age |  |  |  | 0.765 |
| <65 | 657 (50.54) | 0.718 (0.469 ~ 1.100) | 0.128 |  |
| ≥65 | 643 (49.46) | 0.666 (0.492 ~ 0.901) | 0.008 |  |
| HR: Hazard Ratio, CI: Confidence Interval. T2DM, type 2 diabetes mellitus; CHD, coronary heart disease; COPD, chronic obstructive pulmonary disease; AKI, acute kidney injury. | | | | |

| Table S7 Comparison of baseline characteristics of data without imputation after outlier removal | | | | |
| --- | --- | --- | --- | --- |
| Variable Names | Overall | Non-sepsis group | Sepsis group | P value |
| N | 1164 | 984 | 180 |  |
| Age | 63.35±14.89 | 63.14±15.14 | 64.46±13.39 | 0.28 |
| Gender (n, %) |  |  |  | <0.01 |
| Female | 474 (40.72) | 421 (42.78) | 53 (29.44) |  |
| Male | 690 (59.28) | 563 (57.22) | 127 (70.56) |  |
| BMI (kg/m^2^) | 28.27±4.15 | 28.27±4.24 | 28.28±3.65 | 0.96 |
| Hematocrit (n, %) | 35.66±5.87 | 35.91±5.79 | 34.3±6.13 | <0.01 |
| Hemoglobin (g/dL) | 11.85±2.07 | 11.93±2.05 | 11.44±2.16 | <0.01 |
| Platelet (10^9^/L) | 211.51±82.28 | 217.55±80.48 | 178.5±84.37 | <0.01 |
| RDW (%) | 14.08±1.46 | 14±1.43 | 14.51±1.53 | <0.01 |
| RBC (10^12^/L) | 3.9±0.7 | 3.94±0.69 | 3.69±0.73 | <0.01 |
| WBC (10^9^/L) | 9.09±3.9 | 8.79±3.73 | 10.73±4.43 | <0.01 |
| Anion Gap (mEq/L) | 13.4±2.98 | 13.33±2.96 | 13.79±3.05 | 0.06 |
| Calcium Total (mg/dL) | 8.72±0.63 | 8.79±0.59 | 8.34±0.7 | <0.01 |
| Chloride (mEq/L) | 103.07±4.38 | 103.03±4.21 | 103.25±5.23 | 0.55 |
| GLU (mg/dL) | 117.8±34.4 | 116.01±33.65 | 127.59±36.83 | <0.01 |
| Potassium (mEq/L) | 4.06±0.48 | 4.05±0.47 | 4.13±0.53 | 0.05 |
| Sodium (mEq/L) | 138.73±3.44 | 138.84±3.31 | 138.09±4 | 0.01 |
| Creatinine (mg/dL) | 0.94±0.34 | 0.93±0.34 | 1±0.37 | 0.01 |
| Urea nitrogen (mg/dL) | 17.01±8.8 | 16.55±8.51 | 19.56±9.89 | <0.01 |
| Hypertension (n, %) |  |  |  | 0.76 |
| No | 612 (52.58) | 515 (52.34) | 97 (53.89) |  |
| Yes | 552 (47.42) | 469 (47.66) | 83 (46.11) |  |
| T2DM (n, %) |  |  |  | 0.11 |
| No | 904 (77.66) | 773 (78.56) | 131 (72.78) |  |
| Yes | 260 (22.34) | 211 (21.44) | 49 (27.22) |  |
| CHD (n, %) |  |  |  | <0.01 |
| No | 869 (74.66) | 754 (76.63) | 115 (63.89) |  |
| Yes | 295 (25.34) | 230 (23.37) | 65 (36.11) |  |
| COPD (n, %) |  |  |  | 0.07 |
| No | 1038 (89.18) | 885 (89.94) | 153 (85.00) |  |
| Yes | 126 (10.82) | 99 (10.06) | 27 (15.00) |  |
| AKI (n, %) |  |  |  | <0.01 |
| No | 951 (81.70) | 896 (91.06) | 55 (30.56) |  |
| Yes | 213 (18.30) | 88 (8.94) | 125 (69.44) |  |
| 90-day mortality (n, %) |  |  |  | <0.01 |
| Survival | 1092 (93.81) | 942 (95.73) | 150 (83.33) |  |
| Dead | 72 (6.19) | 42 (4.27) | 30 (16.67) |  |
| 365-day mortality (n, %) |  |  |  | <0.01 |
| Survival | 1022 (87.80) | 883 (89.74) | 139 (77.22) |  |
| Dead | 142 (12.20) | 101 (10.26) | 41 (22.78) |  |
| Glucocorticoid (n, %) |  |  |  | <0.01 |
| No | 946 (81.27) | 818 (83.13) | 128 (71.11) |  |
| Yes | 218 (18.73) | 166 (16.87) | 52 (28.89) |  |
| Immunosuppressant (n, %) |  |  |  | 0.78* |
| No | 1138 (97.77) | 961 (97.66) | 177 (98.33) |  |
| Yes | 26 (2.23) | 23 (2.34) | 3 (1.67) |  |
| RDW, red cell distribution width; RBC, red blood cell; WBC, white blood cell; GLU, glucose; BMI, body mass index; T2DM, type 2 diabetes mellitus; CHD, coronary heart disease; COPD, chronic obstructive pulmonary disease; AKI, acute kidney injury. *, Compared using Fisher's exact test. | | | | |

| Table S8 Analysis of the association between calcium and occurrence of sepsis | | | | | | |
| --- | --- | --- | --- | --- | --- | --- |
|  | Model 1 | P value | Model 2 | P value | Model 3 | P value |
| Calcium | 0.303(0.229~0.397) | <0.001 | 0.301(0.227~0.397) | <0.001 | 0.346(0.253~0.468) | <0.001 |
| Q1 | Ref |  | Ref |  | Ref |  |
| Q2 | 0.412(0.273~0.615) | <0.001 | 0.410(0.271~0.614) | <0.001 | 0.456(0.293~0.701) | <0.001 |
| Q3 | 0.184(0.110~0.298) | <0.001 | 0.184(0.109~0.297) | <0.001 | 0.220(0.128~0.366) | <0.001 |
| Q4 | 0.172(0.100~0.282) | <0.001 | 0.181(0.105~0.298) | <0.001 | 0.223(0.125~0.384) | <0.001 |
| P for trend |  | <0.001 |  | <0.001 |  | <0.001 |
| Model 1: no covariates were adjusted. Model 2: age and gender were adjusted. Model 3: age, gender, platelet, white blood cell, urea nitrogen, glucocorticoid and anion gap were adjusted. | | | | | | |

| Table S9 Analysis of the Association Between Calcium and all-cause Mortality | | | | | | |
| --- | --- | --- | --- | --- | --- | --- |
|  | Model 1 | P value | Model 2 | P value | Model 3 | P value |
| 90-day mortality |  |  |  |  |  |  |
| Calcium | 0.602 (0.419 ~ 0.864) | 0.006 | 0.543 (0.373 ~ 0.792) | 0.001 | 0.577 (0.389 ~ 0.854) | 0.006 |
| Q1 | Ref |  | Ref |  | Ref |  |
| Q2 | 0.540 (0.291 ~ 1.004) | 0.051 | 0.500 (0.269 ~ 0.931) | 0.029 | 0.467 (0.245 ~ 0.891) | 0.021 |
| Q3 | 0.481 (0.255 ~ 0.908) | 0.024 | 0.452 (0.240 ~ 0.854) | 0.014 | 0.514 (0.269 ~ 0.983) | 0.044 |
| Q4 | 0.460 (0.240 ~ 0.883) | 0.019 | 0.411 (0.213 ~ 0.793) | 0.008 | 0.410 (0.205 ~ 0.823) | 0.012 |
| P for trend |  | 0.021 |  | 0.009 |  | 0.022 |
| 365-day mortality |  |  |  |  |  |  |
| Calcium | 0.641 (0.495 ~ 0.831) | 0.010 | 0.585 (0.447 ~ 0.765) | <0.001 | 0.598 (0.452 ~ 0.792) | <0.001 |
| Q1 | Ref |  | Ref |  | Ref |  |
| Q2 | 0.707 (0.461 ~ 1.083) | 0.111 | 0.664 (0.433 ~ 1.018) | 0.061 | 0.635 (0.409 ~ 0.986) | 0.043 |
| Q3 | 0.515 (0.324 ~ 0.818) | 0.005 | 0.486 (0.306 ~ 0.773) | 0.002 | 0.521 (0.325 ~ 0.835) | 0.007 |
| Q4 | 0.532 (0.334 ~ 0.845) | 0.008 | 0.479 (0.300 ~ 0.765) | 0.002 | 0.476 (0.290 ~ 0.780) | 0.003 |
| P for trend |  | 0.003 |  | 0.001 |  | 0.002 |
| Model 1: no covariates were adjusted. Model 2: age and gender were adjusted. Model 3: age, gender, platelet, white blood cell, urea nitrogen, glucocorticoid and anion gap were adjusted. | | | | | | |

| Table S10 Platelet as a mediator variable between Calcium and the correlation of 365-day mortality | | | | |
| --- | --- | --- | --- | --- |
| Mediation effect | Estimate | 95% CI Lower | 95% CI Upper | P value |
| Total effect | 21.818 | 1.876 | 347.47 | <0.001 |
| ACME | 5.524 | 0.122 | 162.57 | <0.001 |
| ADE | 16.293 | 1.151 | 172.27 | 0.02 |
| Proportion mediated | 0.253 | 0.065 | 0.57 | <0.001 |
| ACME, average causal mediation effect, ADE, average direct effect. | | | | |

| Table S11 Analysis of the association between calcium and occurrence of sepsis | | | | | | |
| --- | --- | --- | --- | --- | --- | --- |
|  | Model 1 | P value | Model 2 | P value | Model 3 | P value |
| Calcium | 0.277(0.205~0.372) | <0.001 | 0.278(0.204~0.376) | <0.001 | 0.336(0.238~0.468) | <0.001 |
| Q1 | Ref |  | Ref |  | Ref |  |
| Q2 | 0.361(0.227~0.562) | <0.001 | 0.358(0.225~0.560) | <0.001 | 0.451(0.275~0.729) | 0.001 |
| Q3 | 0.192(0.111~0.318) | <0.001 | 0.192(0.111~0.319) | <0.001 | 0.241(0.135~0.414) | <0.001 |
| Q4 | 0.136(0.072~0.240) | <0.001 | 0.148(0.078~0.261) | <0.001 | 0.205(0.104~0.380) | <0.001 |
| P for trend |  | <0.001 |  | <0.001 |  | <0.001 |
| Model 1: no covariates were adjusted. Model 2: age and gender were adjusted. Model 3: age, gender, platelet, white blood cell, urea nitrogen, glucocorticoid and anion gap were adjusted. | | | | | | |

| Table S12 Analysis of the Association Between Calcium and all-cause Mortality | | | | | | |
| --- | --- | --- | --- | --- | --- | --- |
|  | Model 1 | P value | Model 2 | P value | Model 3 | P value |
| 90-day mortality |  |  |  |  |  |  |
| Calcium | 0.618 (0.419 ~ 0.911) | 0.015 | 0.565 (0.377 ~ 0.848) | 0.006 | 0.643 (0.416 ~ 0.995) | 0.047 |
| Q1 | Ref |  | Ref |  | Ref |  |
| Q2 | 0.479 (0.238 ~ 0.966) | 0.040 | 0.449 (0.223 ~ 0.907) | 0.026 | 0.489 (0.236 ~ 1.013) | 0.054 |
| Q3 | 0.475 (0.241 ~ 0.938) | 0.032 | 0.439 (0.222 ~ 0.869) | 0.018 | 0.530 (0.263 ~ 1.068) | 0.076 |
| Q4 | 0.451 (0.224 ~ 0.909) | 0.026 | 0.416 (0.205 ~ 0.845) | 0.015 | 0.473 (0.219 ~ 1.019) | 0.056 |
| P for trend |  | 0.037 |  | 0.023 |  | 0.081 |
| 365-day mortality |  |  |  |  |  |  |
| Calcium | 0.595 (0.450 ~ 0.786) | <0.001 | 0.538 (0.402 ~ 0.719) | <0.001 | 0.572 (0.419 ~ 0.780) | <0.001 |
| Q1 | Ref |  | Ref |  | Ref |  |
| Q2 | 0.677 (0.425 ~ 1.078) | 0.100 | 0.635 (0.398 ~ 1.012) | 0.056 | 0.663 (0.409 ~ 1.075) | 0.096 |
| Q3 | 0.501 (0.305 ~ 0.824) | 0.006 | 0.458 (0.278 ~ 0.755) | 0.002 | 0.510 (0.306 ~ 0.851) | 0.010 |
| Q4 | 0.427 (0.251 ~ 0.726) | 0.002 | 0.386 (0.226 ~ 0.660) | <0.001 | 0.405 (0.228 ~ 0.719) | 0.002 |
| P for trend |  | <0.001 |  | <0.001 |  | 0.001 |
| Model 1: no covariates were adjusted. Model 2: age and gender were adjusted. Model 3: age, gender, platelet, white blood cell, urea nitrogen, glucocorticoid and anion gap were adjusted. | | | | | | |

| Table S13 Platelet as a mediator variable between Calcium and the correlation of 365-day mortality | | | | |
| --- | --- | --- | --- | --- |
| Mediation effect | Estimate | 95% CI Lower | 95% CI Upper | P value |
| Total effect | 10.767 | 0.580 | 222.30 | <0.001 |
| ACME | 3.187 | 0.067 | 113.32 | <0.001 |
| ADE | 7.581 | 0.429 | 103.88 | <0.001 |
| Proportion mediated | 0.296 | 0.090 | 0.64 | <0.001 |
| ACME, average causal mediation effect, ADE, average direct effect. | | | | |
